# Comparing Five Major Knee Osteoarthritis Cohort Studies: Similarities, Differences, and Unique Aspects of CHECK, OAI, FNIH, IMI-APPROACH, and MOST

**DOI:** 10.1177/19476035251326276

**Published:** 2025-03-19

**Authors:** Mariia Oliinyk, Simon C. Mastbergen, Anne Karien C. A. Marijnissen, David T. Felson, David J. Hunter, Micheal C. Nevitt, Harrie Weinans, Mylène P. Jansen

**Affiliations:** 1 Department of Rheumatology & Clinical Immunology, University Medical Center Utrecht, Utrecht, The Netherlands; 2 Department of Internal Medicine No. 3 and Endocrinology, Kharkiv National Medical University, Kharkiv, Ukraine; 3 School of Medicine, Boston University, Boston, MA, USA; 4 Sydney Musculoskeletal Health, Kolling Institute, The University of Sydney, Sydney, NSW, Australia; 5 Rheumatology Department, Northern Sydney Local Health District, Sydney, NSW, Australia; 6 Department of Epidemiology & Biostatistics, University of California, San Francisco, San Francisco, CA, USA; 7 Department of Orthopedics, University Medical Center Utrecht, Utrecht, The Netherlands; 8 Department of Biomechanical Engineering, TU Delft, Delft, The Netherlands

**Keywords:** knee osteoarthritis, cohort, observational, multicenter, narrative review

## Abstract

**Objective:**

To analyze and synthesize the information available from five pivotal, large-scale, multicenter, observational studies (CHECK, OAI, FNIH Biomarkers Consortium, IMI-APPROACH, and MOST) focusing on knee osteoarthritis (OA), which can be used to elucidate disease progression, risk factors, and the effectiveness of potential interventions.

**Design:**

For this narrative review, a comprehensive literature search and data extraction from official web pages and scientific databases were conducted to compare methodologies, in- and exclusion criteria, outcomes, and cohort characteristics across the studies. Thematic, comparative, and qualitative analyses were employed to identify trends, commonalities, and disparities among the findings.

**Results:**

The studies collectively enhanced understanding of the onset and progression of knee OA, and in several of the studies, hip OA, emphasizing the importance of both systemic and local risk factors. Advanced imaging and biomarkers are important components in all the cohorts, with the goal of aiding early diagnosis and tracking disease progression. All cohorts evaluated unique markers generally not available in the other cohorts, while other factors overlap, suggesting possibilities for combining or cross-validating between cohorts.

**Conclusions:**

The collaborative efforts of major OA research significantly advance our understanding of knee OA. These studies highlight the importance of a multifaceted approach, integrating advanced imaging, biomarkers, and longitudinal data to tackle the complexities of OA. By synthesizing findings and addressing knowledge gaps such as heterogeneity of patients and used measurements, and use of novel pain measures, future research can develop more effective diagnostic tools and treatments, ultimately enhancing the quality of life for OA patients.

## Introduction

Osteoarthritis (OA) is a prevalent joint disease that affects millions worldwide, most commonly affecting the knee joint and leading to pain, disability, and decreased quality of life.^1-3^ Despite its prevalence, the exact causes of OA remain only partially understood. This complexity has stimulated numerous research initiatives aimed at understanding the mechanisms of OA development, improving diagnosis, and developing effective treatments.

Significant among these efforts are different large-scale, observational, longitudinal studies, each contributing unique insights into knee OA’s onset, progression, risk factors, and potential interventions. The five major multicenter knee OA studies with publicly available data include the Cohort Hip and Cohort Knee (CHECK) study,^4^ the Osteoarthritis Initiative (OAI),^5,6^ Foundation for the National Institutes of Health (FNIH) Biomarkers Consortium,^7^ Innovative Medicine Initiative Applied Public-Private Research enabling OsteoArthritis Clinical Headway (IMI-APPROACH),^8^ and Multi-center Osteoarthritis Study (MOST).^9^ Collectively, these studies represent the concerted effort of the global scientific community to tackle OA from multiple angles.

These studies, with their diverse approaches, measurements, and foci, reflect the complexity of OA and the multi-faceted strategy required to combat it. They leverage advancements in imaging, biomarker research, and clinical epidemiology, offering hope for OA prevention, diagnosis, and treatment breakthroughs.

We compare these research efforts to identify commonalities, differences, and trends across various studies. This comparison identifies opportunities to synthesize findings, to cross- validate results, pool data for analyses and address gaps in knowledge. In addition, comparing research helps in understanding the strengths and limitations of different study designs, methodologies, and data collection techniques, and can aid researchers with designing their studies and selecting a cohort for their research questions. Ultimately, this comparative analysis aids in advancing our understanding of OA research and in guiding future research directions.

## Methods

For this narrative review, a literature search for full-text articles published by the CHECK, OAI, IMI-APPROACH, FNIH, and MOST was conducted. This search used the official web pages of these research entities and databases such as PubMed, Web of Science, Scopus, and Google Scholar. Our task is to compare these studies and identify similarities and differences between them, to pinpoint any gaps that need addressing for future OA research questions. We employed data extraction to receive the necessary information from each study. We used thematic analysis to identify common themes, such as risk factors, biomarkers, and imaging methods, across the studies. The comparative analysis highlighted key differences and similarities in cohort designs, inclusion criteria, and methodologies.

The objectives, design, methods, and measurement employed in the five initiatives are summarized below.

*The CHECK Study* was initiated in The Netherlands, starting inclusion of participants in 2002 to understand the natural progression of early symptomatic OA in the hip and knee. Participants aged 45 to 65 years with hip or knee pain, without a previous healthcare consultation or with first consultation <6 months ago for these complaints to their primary care physician, were included. Participants were divided into two groups based on symptom severity and were followed with regular visits (based on symptoms severity) to the research center for up to 10 years. This approach allows researchers to monitor the course and prognosis of early osteoarthritis and understand its underlying mechanisms.^4^

*The OAI* was launched in 2002 as a public-private partnership between the National Institutes of Health (NIH) and several pharmaceutical companies focusing on knee OA, and secondarily on hip and hand OA. It aimed to identify risk factors and imaging and biochemical biomarkers for the development and progression of knee OA by enrolling individuals who either had symptomatic radiographic knee OA or were at increased risk of developing it based on the presence of risk factors. The study’s design facilitates a deep understanding of OA’s natural history and the evaluation of potential biomarkers for early diagnosis and monitoring disease progression across a broad range of baseline disease severity.^5,6^

*FNIH Osteoarthritis Biomarkers Study* focused on stratifying participants into distinct structural phenotypes based on semiquantitative assessment to determine the risk for pain and structural progression over 48 months. The study selected participants who had symptomatic radiographic knee OA from those already enrolled in the OAI using a nested case-control study design with the case and control groups of participants based on the presence and absence of radiographic and pain progression during the first two years of the OAI. The project focused on validating imaging and biochemical biomarkers for knee OA progression, critical for monitoring disease progression and response to treatment.^7^

*IMI-APPROACH* was a European initiative that included patients with tibiofemoral OA from the five European observational OA cohorts, starting in 2018, to create a detailed OA phenotyping framework. Machine learning (ML) models developed on data from the CHECK cohort were used for patient selection, including patients with a higher predicted chance of structural and/or pain progression. The study aimed to validate whether these patients with higher probabilities for progression experienced disease progression, to facilitate targeted drug therapy.^8^

*MOST* focused on understanding the risk factors for OA development and progression. MOST included older Americans with OA disease or at increased risk of developing it, assessing pain, function, and structural changes in the knee over time. These data allow for a comprehensive evaluation of the natural history of knee OA and the identification of potential interventions to prevent or slow its progression. The study’s aim was to identify novel and modifiable biomechanical factors, bone and joint structural factors, and nutritional factors that affect the occurrence and progression of OA-related knee symptoms and radiographic knee OA.^9^

### Cohort Inclusion and Exclusion Criteria

The common thing of all studies is the focus on knee OA: Except for *CHECK*, which includes hip complaints as well, all studies had a primary focus solely on knee OA. Some studies included participants based on OA risk factors such as overweight, history of knee injury or surgery, and family history of knee replacement, acknowledging these as significant contributors to OA development. There is a general trend toward including older adults, typically starting at 45 or 50 years of age, recognizing that OA prevalence increases with age. All inclusion and exclusion criteria are presented in Table S1.

The cohorts differed on inclusion criteria: *CHECK* targeted a younger demographic (45–65 years) with recent nontraumatic knee or hip pain/stiffness and no prior extensive consultation, aiming to investigate OA at an early stage.^4^ *OAI* included overweight individuals or those with a history of knee injury/surgery, or a family history of knee replacement, emphasizing structural risk factors for OA. *FNIH* focused on OAI participants with specific Kellgren-Lawrence grade (K&L) grades and required baseline imaging, selecting individuals with established but varied severity of knee OA for biomarker identification. *IMI-APPROACH* selected participants based on the clinical ACR classification criteria for knee OA and a high probability of progression, targeting a population with established clinical OA likely to show disease progression.^8^ *MOST* involved participants with frequent knee pain or those at risk due to weight, injury history, or surgery, aiming to study OA progression and incidence in a broadly at-risk population.^9^

Unique aspects include inclusion from an ongoing cohort for the *FNIH* and inclusion based on predicted knee OA progression for *IMI-APPROACH*. The exclusion criteria across the studies present both similarities and differences. Common exclusion criteria are the presence of other rheumatic diseases, recent joint replacements of index knee, and major joint surgery, severe comorbidities (e.g., end-stage renal disease in *MOST* or malignancy within the past 5 years in *CHECK*), and mobility issues (inability to walk without assistance can limit the possibility to complete physical evaluations).

Each study had several unique or study-specific exclusion criteria. *CHECK* excluded conditions like congenital dysplasia, osteochondritis dissecans, or ligament/meniscus damage that could explain symptoms, aiming for a clear diagnosis of OA. In addition, comorbidity precluding a long-term follow-up of at least 10 years and inability to understand Dutch were exclusion criteria, reflecting the study’s geographic and logistical considerations. For *OAI*, inclusion was relatively broad and criteria were limited, but like *IMI-APPROACH* exclusion criteria included the inability to undergo MRI and/or CT, taking into account these studies focus on imaging-based risk factors and outcomes. *FNIH* OA Biomarkers Consortium used specific radiographic and pain criteria for exclusion, excluding knees with KL 4 or medial joint space width <1.0 mm and little pain (based on WOMAC (The Western Ontario and McMaster Universities Arthritis Index), to select participants with room for measurable disease progression. For *IMI-APPROACH*, participants with patellofemoral OA, secondary knee OA, and those with various specific conditions (e.g., severe chondrocalcinosis, visual leg deformity) or potential for pregnancy were excluded, reflecting a detailed and specific participant profile for study interventions and imaging requirements.

Each study’s inclusion, and exclusion criteria were tailored to its specific research goals, whether that was understanding early OA development, identifying biomarkers for progression, or assessing the efficacy of potential treatments across diverse populations with varying stages of OA.

## Results

### Eligibility, Recruitment, and Demographics

#### CHECK

Ten hospitals, both general and university-affiliated, situated in semi-urban areas of The Netherlands, included patients from October 2002 to September 2005. General practitioners near the participating hospitals were asked to refer eligible individuals. In addition, recruitment efforts included advertisements and articles in local newspapers and on the Dutch Arthritis Foundation website. A group of 1,002 individuals formed the cohort and were monitored for 10 years. The mean age of included patients was 56 years old and 79% of patients were female.^4^ CHECK data can be accessed through DANS Data Station Life Sciences (https://doi.org/10.17026/dans-xs3-ws3s).

#### OAI

The OAI was initiated in 2002, and supported by the collaboration of the NIH, the private sector, and other funding entities (ClinicalTrials.gov Identifier: NCT00080171). From March 2004 to May 2006, four clinical centers in the United States (University of Maryland, Brown University, and University of Pittsburgh, Ohio State University) recruited and monitored participants with knee OA or those at high risk of developing knee OA for an 8-year duration. The total number of patients was 4,796. They were divided into two subcohorts: one with symptomatic radiographic knee osteoarthritis (defined as having frequent knee symptoms and radiographic TF knee osteoarthritis, defined as K&L≥2; progression cohort), and a second cohort of 3,285 individuals without symptomatic knee osteoarthritis, selected based on specific characteristics indicating an increased risk of developing symptomatic knee osteoarthritis (incidence cohort).^10^ All patients were aged between 45 and 79 years, with 59% being women and representing various ethnic backgrounds.^6^ All details and data of the OAI are available on the internet (https://nda.nih.gov/oai).

#### FNIH

The FNIH Osteoarthritis Biomarkers Consortium study was a nested case-control study embedded within the larger OAI study.^11^ The research took place between 2012 and 2014, involving a total of 600 patients. They were categorized into two main outcome groups, each with one study knee per patient. One group consisted of knees showing clinically relevant progression, both in terms of radiographic changes and pain (*n* = 194; cases), while the other group comprised OA knees without the combination of radiographic and pain progression, serving as controls (*n* = 406). Considering that the available data from this study are categorized into two subgroups, we have preserved this division for the patients in our tables. FNIH data can be accessed through the OAI database (https://nda.nih.gov/oai).

#### IMI-APPROACH

Follow-up of the 297 patients included lasted for 2 years. Because the existing source cohorts could not all provide sufficient patients due to the selection process, patients withdrawing consent, and noncompliance with inclusion criteria, a small number of additional patients were recruited from outpatient departments and invited for a screening visit. Individuals with predominant tibiofemoral OA were identified from five European observational OA cohorts (CHECK, HOSTAS, MUST, PROCOAC, and DIGICOD) through an ML technique trained on longitudinal data from the CHECK cohort and adapted for specific cohorts using available data from each. The average age was 66.5 years and the majority of patients were women—77.44%. From 432 screened patients, those with a high likelihood of structural progression and/or pain progression over two years were identified, using two ML models designed to assess the probability of each patient being a “progressor.” Structural progression was defined as a decrease in JSW of ≥0.3 mm per year over 2–3 years (0.7 mm being the minimal detectable difference in radiographic JSW). Pain progression (Knee injury and Osteoarthritis Outcome Score (KOOS) pain on a 0–100 scale) was defined as at least one of the following: fast/significant pain increase and/or stable significant pain. Fast pain increase: KOOS pain decrease between baseline and follow-up ≥10 points per year (i.e. ≥20 points decrease in the 2-year follow-up period) and final KOOS pain score ≤65 points. Significant pain increase: KOOS pain decrease between baseline and follow-up ≥5 points per year (i.e. ≥10 points decrease in the 2-year follow-up period) and final KOOS pain score ≤60 points. Stable significant pain: KOOS pain score ≤60 points during the whole study period. IMI-APPROACH data access details can be found online (https://datacatalog.elixir-luxembourg.org/e/project/dc9a4cc0-147a-11eb-b51f-8c8590c45a21).

#### MOST

The MOST Study, funded by the NIH, is a longitudinal observational research effort involving initially 3026 men (MOST Original Cohort) and women aged 50 to 79 living in communities, who either had knee OA or exhibited known risk factors for knee OA, such as age, female sex, overweight, and a history of knee symptoms, injury, or surgery.^12,13^ Participant recruitment and measurements were conducted at the clinical centers of the University of Alabama at Birmingham (UAB) and The University of Iowa (UI). These centers aimed to enroll a community-based sample of men and women, ensuring a demographic representation that mirrors the age and sex distribution of the U.S. population. Specifically, participants were drawn from the general population but are selected to have either existing knee OA (one-third) or a high risk for knee OA (two-thirds).^14^ While the original MOST cohort followed participants for 7 years, an additional 1,500 subjects aged 45 to 69 with no OA or mild OA and not troubled by severe knee pain were recruited for 12-year follow-up (MOST New Cohort). The data presented in this article is accurate for the MOST Original Cohort. MOST details and data can be found online (https://agingresearchbiobank.nia.nih.gov/studies/most/).

The baseline characteristics for all participants of the five cohorts are presented in Table 1.

**Table 1.** Cohort Demographics and Disease Characteristics.

	CHECK	OAI (Combined Incidence and Progression Cohort)	FNIH OA Biomarkers Consortium		IMI-APPROACH	MOST (Multicenter Osteoarthritis Study)
Location	Europe	United States	United States /Australia		Europe	United States
Number of patients	1002	4674	194	406	297	3026
Inclusion period	2002–2005	2004–2006	2018		2018–2019	2003–2005
Years of follow-up	10	10	4		2	7
Type of OA (primary focus)	Knee, Hip	Knee	Knee		Knee	Knee
Age in years (mean (SD))	56 (5)	61.3 (9.2)	62.0 (8.8)	61.3 (8.9)	66.5 (7.1)	62.5 (8.1)
Sex, female	79%	58%	56.7%	59.9%	77.44%	60.1%
BMI, kg/m^2^ (mean (SD) or mean)	26 (4)^a^	28.9 (4.8)	30.7 (4.8)	30.7 (4.8)	28.1	30.7
Pain Score (mean (SD) or mean)	WOMAC 25 (17)^a^	WOMAC 2.5 (3.4)	WOMAC 10.2 (12.7)	WOMAC 12.5 (16.3)	KOOS 31.3	WOMAC, KOOS 2.40 (2.97)
K&L						
Grade 0	68%	17%	0%	0%	17%	46.9% K/L grade ≤ 1
Grade 1	25%	36%	12.4%	12.6%	30%	
Grade 2	6%	20%	43.3%	54.7%	30%	53.1% K/L grade ≥ 2
Grade 3	1%	21%	44.3%	32.8%	18%	
Grade 4	NA	6%	0%	0%	3%	

CHECK = Cohort Hip and Cohort Knee; OAI = Osteoarthritis Initiative; FNIH = Foundation for the National Institutes of Health; IMI-APPROACH = Innovative Medicine Initiative Applied Public-Private Research enabling OsteoArthritis Clinical Headway; MOST = Multi-center Osteoarthritis Study; OA = osteoarthritis; BMI = body mass index; WOMAC = Western Ontario and McMaster Universities Osteoarthritis Index; K&L = Kellgren & Lawrence.

a 2% to 3% missing (145 subjects were lost to follow-up among 1,002).

**Figure 1.**
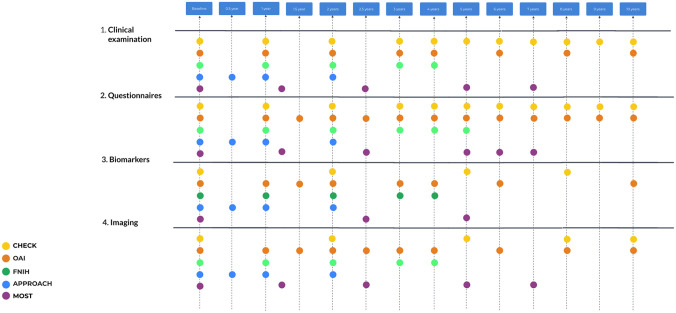
Overall investigations schedule. For OAI and MOST, biomarker specimens are available, but no measurements were performed. OAI = Osteoarthritis Initiative; MOST = Multi-center Osteoarthritis Study.

All studies include an extensive list of data, including clinical examination and self-assessment using various questionnaires, collection of blood and urine samples, as well as different imaging studies. The timing of data collection differs across studies. An overview of these particulars is outlined in **Fig. 1**.

The main time points for collection data for CHECK are baseline (BL), 1, 2, 3, 4, 5, 6, 7, 8, 9, 10 years;^4^ for OAI: BL, 12, 18, 24, 30, 48, 60, 72, 84, 96, 108, 120/132 months (https://nda.nih.gov/); for FNIH BL, 12, 24, 36, 48;^15^ for IMI-APPROACH: BL, 6, 12, 24 months;^8^ for MOST: BL, 15, 30, 60 (cycle 2 BL), 72 and 84 months.^14^

### Clinical Joint Examination

**Figure 2.**
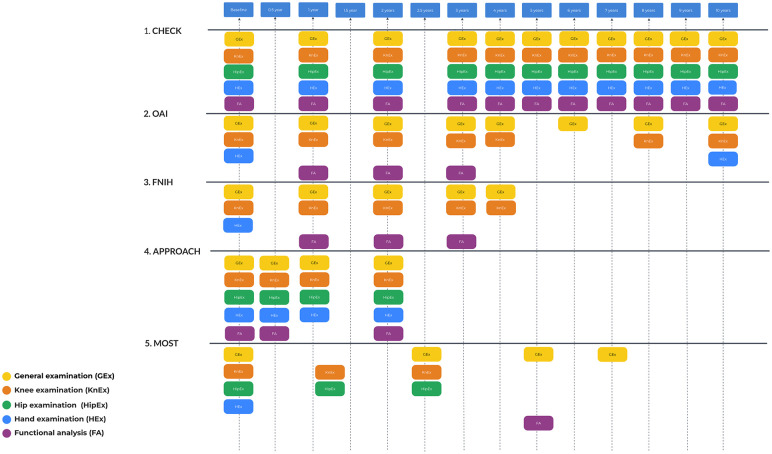
Clinical examination investigation schedule. There are no additional examinations for FNIH compared with OAI. FNIH = Foundation for the National Institutes of Health; OAI = Osteoarthritis Initiative.

In OA studies, physical examination predominantly focuses on the joints most commonly affected by this disease. These typically include knee joints. Hip OA is another common focus, especially in studies like CHECK, which explicitly includes Cohort Hip in its name, and more rarely includes other facilities for examination like hands, fingers, spine, feet, and ankles (**Fig. 2**).

#### CHECK

Knee, hip, and hand joints: The examination of knees and hips joints included assessments of pain, range of motion, palpable warmth, bony tenderness, joint swelling, and functional assessments specific to these joints. Hand joint examination included a range of motion and joint swelling, but also DIP/PIP, and CMC I bony enlargements.^4^

#### OAI

Knee joints: The OAI focused extensively on the knee, performing detailed evaluations that include alignment (by goniometer), anserine bursa tenderness, effusion, range of motion, tibiofemoral joint line tenderness, knee flexion pain/tenderness, patellar tenderness, patellar quadriceps tenderness/tendinitis, patellofemoral crepitus, medial-lateral laxity, knee pain location (knee pain map). In addition, an examination of the hand joints was carried out, which included DIP bony enlargements.

#### FNIH

The FNIH Osteoarthritis Biomarkers Consortium study was a case-control study situated within the broader framework of the OAI study, so it did not include additional physical examination.^15^

#### IMI-APPROACH

Knee, hip, and hand joints: Knee examinations included warmth of the knee, effusion (positive patellar tap), passive ranges of flexion and extension, presence of a flexum, presence of varus or valgus, and pain and grinding in the patellofemoral joint (grinding test). Hip examinations included a passive range of motions and the presence of a flexum. Hand joint examination included osteophytosis and inflammation of all hand joints, deformity of CMC-1, DIP/PIP 2-3 and MCP-1, and Doyle articular index.

#### MOST

Like the OAI, MOST focused significantly on knee OA but also examined other joints that could be at risk in the older population, such as hip and hands. At enrollment, 15 months, and 30 months, physical examinations of joints were carried out. These assessments included knee range of motion and evaluations of tenderness at specific sites such as the greater trochanters, iliotibial band at the lateral femoral condyle, anserine bursa, tibiofemoral joint line, lateral and medial patella, medial knee fat pad, trapezius, and lateral epicondyle. In addition, hip internal rotation (both pain and range of motion) was examined. Proprioceptive acuity was evaluated by measuring nonweightbearing joint reposition sense.^13^

All the timing of the physical examination, as well as the joints included in the examination, can be seen in **Figure 2**.

Some research included specific investigation of gait, namely IMI-APPROACH and MOST. The motion analysis in IMI-APPROACH was performed with the GaitSmart^TM^ system. The GaitSmart^TM^ system uses six inertial measurement units, comprising 3 tri-axial accelerometers and three tri-axial gyroscopes. Investigation was made at BL, 6 and 24 months.^16,17^ In the MOST study foot loading and gait parameters were evaluated using an Emed-X digital pedobarograph (Novel Electronics, Inc., St. Paul, MN) and a GAITRite walkway (MAP/CIR Systems, Inc., Havertown, PA), respectively. Participants underwent four trials each of usual and fast-paced walking to gather gait parameters, and five trials of usual-paced walking to collect plantar pressure data for each foot. This investigation was done on month 60 (**Fig. 2**).^14^ The OAI and MOST studies included the use of accelerometers to monitor physical activity, with the OAI doing so at the 4-year visit and MOST at the 5-year and 7-year visits.^9,18^

Unlike the other cohorts, the MOST study included quantitative sensory testing (QST) to analyze pain sensitization. Parameters included patients’ pain pressure threshold (PPT) and mechanical temporal summation (TS).

### Questionnaires

A summary of topics assessed by questionnaires in each study can be found in **Table S2** .

#### CHECK

**Figure 3.**
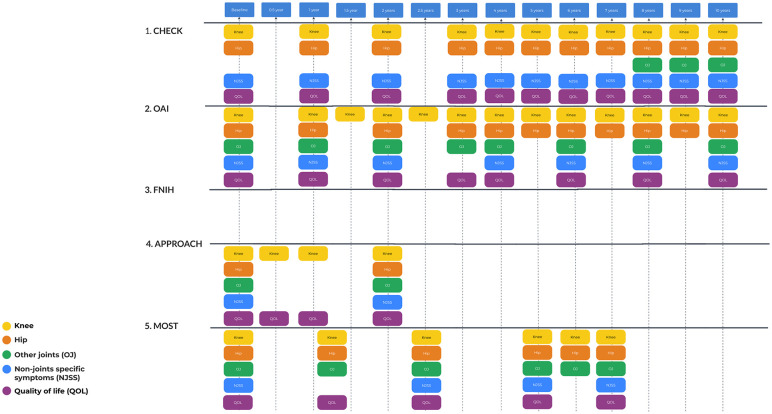
Questionnaire investigation schedule. There are no additional questionnaires for FNIH compared with OAI. FNIH = Foundation for the National Institutes of Health; OAI = Osteoarthritis Initiative.

The self-reported questionnaires are designed to evaluate symptoms in the hips and knees,^19,20^ hands,^21^ and the severity of pain.^22^ They also assess coping strategies,^23^ health-related quality of life,^24-26^ leisure activities and employment,^27^ economic consequences,^28^ social support,^29^ and comorbidities.^29^ The schedule for the questionnaires is presented in **Fig. 3**.

#### OAI

Self-reported questionnaires were used to evaluate knee symptoms, pain, physical activity, quality of life, comorbidities, coping, depressive symptoms, and cognition.

#### FNIH

No additional questionnaires compared with OAI.

#### IMI-APPROACH

Knee, hip, and hand symptoms were evaluated with self-reported questionnaires, as well as pain and quality of life.

#### MOST

Self-reported questionnaires were used to assess knee and hip symptoms, pain, physical activity, quality of life, comorbidities, coping, depressive symptoms, cognition, and sleep quality.

In all cohorts, WOMAC and KOOS were used to evaluate knee symptoms, signifying a standardized approach in OA research. The Pain NRS (Numeric Rating Scale) was used in all cohorts to assess pain intensity. ICOAP (Measure of Intermittent and Constant Osteoarthritis Pain) was frequently used as well (CHECK, IMI-APPROACH, and MOST), indicating its importance in capturing the unique characteristics of pain. To assess general health and quality of life, highlighting their relevance in understanding the broader impacts of OA on daily living commonly used the SF (Short Form) health survey versions (SF-36, SF-12).

As for differences, only IMI-APPROACH (HOOS (Hip Injury and Osteoarthritis Outcome Score), MOST (WOMAC), and CHECK (NRS pain) assessed hip symptoms, whereas AUSCAN (Australian/Canadian Hand Osteoarthritis Index) and FIHOA (Functional Index for Hand OsteoArthritis) were specific to hand symptomatic assessments in CHECK and IMI-APPROACH, respectively, showing tailored approaches to different joints affected by OA. Physical activity and comorbidity were assessed with PASE scores and comorbidity indices specifically in the OAI Incidence Cohort and MOST, suggesting a focus on the role of physical activity in OA progression and management. Mental health and coping tools for assessing coping mechanisms, depressive symptoms, and cognition show significant variation across the studies. For example, the Coping Strategies Questionnaire and Pain Catastrophizing Scale are used in different combinations, reflecting varied interests in psychological aspects of OA management. Depressive symptoms are measured using the CES-D (Center for Epidemiologic Studies Depression Scale) in the OAI Incidence Cohort and MOST, pointing to a shared concern for mental health in OA.

Each study incorporates some unique measures, such as the Pittsburgh Sleep Quality Index in MOST, the PainDETECT questionnaire for neuropathic pain detection in IMI-APPROACH, and various cognitive screening tools across studies, highlighting specific areas of interest or concern within each cohort.

### Biomarkers

**Figure 4.**
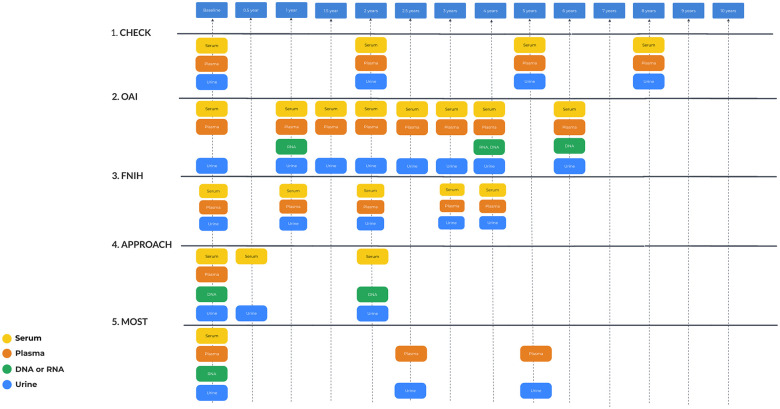
Biomarker investigations schedule.

Of the five cohorts, CHECK, FNIH, and IMI-APPROACH evaluated biomarkers from blood/urine, while OAI and MOST did not. **Fig. 4.** displays the timepoints of blood and urine sample collection. Specimens are available in OAI and MOST to assess biomarkers.

#### CHECK

The selection of biochemical markers for assessment at baseline in the CHECK study was informed by a systematic review of available literature on biochemical markers in knee and hip osteoarthritis (OA).^19^ Blood and urine samples have been gathered from every participant according to a standardized procedure across all sites. Eleven biomarkers were selected to provide a comprehensive representation of joint metabolism, encompassing both anabolic and catabolic pathways in cartilage, (subchondral) bone, and synovial tissue, aligning with current knowledge.^30^ Given the uncertain role of adipokines in OA pathogenesis,^31-34^ three adipokines were additionally evaluated^35^ ( **Table S3** ).

#### FNIH

18 Biomarkers were chosen for this research ( **Table S3** ), reflecting both the breakdown (catabolic) and building (anabolic) activities of cartilage and bone. Some biomarkers were assessed in both serum and urine samples. LabCorp Clinical Trials, certified under Clinical Laboratory Improvement Amendments (CLIA) and College of American Pathologists (CAP), conducted all biomarker assays, except for urine Col2-1 NO2, which was analyzed by Artialis, a facility certified under Good Laboratory Practice (GLP). All assays were carried out in a blinded manner, ensuring no access to clinical information during analysis.^36^

#### IMI-APPROACH

The selection of biomarkers was informed by the current understanding of joint tissue turnover and OA. At baseline, serum and urine samples were obtained for the analysis of 16 biochemical markers. These biomarkers were assessed in laboratories certified by the International Organization for Standardization (ISO) at Nordic Bioscience and Lund University.^37^

Common biomarkers in the cohorts: pAdiponectin, pLeptin, and pResistin are adipokines recognized across the studies, reflecting the importance of metabolic factors in OA. For cartilage degradation and synthesis: sCOMP and sCS846 were consistently used, highlighting the focus on cartilage turnover as a central theme in OA research. Furthermore, hyaluronic acid (HA): sHA is a common marker, utilized for its role in indicating synovial inflammation and cartilage degradation. Collagen breakdown products sC1, 2C, and uCTX-II were consistently analyzed as well, emphasizing the significance of collagen degradation in OA.

As for differences among the consortia CHECK used specific biomarkers as sOC (osteocalcin), sPIIANP, sPIIINP, and sPINP. Urine markers included uCTX-I and uNTX-I pointing toward its interest in bone resorption alongside cartilage degradation. FNIH specific biomarkers included detailed cartilage turnover markers: sC2C, sColl2-1 NO2, sCPII, and sNTXI. IMI-APPROACH used a broad spectrum of molecular pathway markers such as sARGS, sC2M, sC3M, and sNMID, to cover various aspects of OA including inflammation, cartilage integrity, and turnover. A list of all used biomarkers is presented in **Table S3** and a schedule of blood and urine samples is in **Figure 4**.

### Imaging

#### CHECK

K&L grading (ranging from 0 to 4), based on weight-bearing posterior-anterior views of the knees and the anterior-posterior views of the pelvis in radiographs was conducted.^38^ In addition, specific characteristics of the knee and hip are evaluated on other radiographic images according to the OARSI atlas set forth by Altman and Gold.^39^ and the radiographic atlas developed by Burnett *et al.*, which use a scale of 0 to 3.^40^ These evaluations were carried out by five trained specialists who reviewed the radiographs in sequence, aware of their order but not the clinical details of the subjects. In a study of 38 individuals whose radiographs were assessed by all five reviewers, the interobserver agreement was found to be moderate to substantial (with a kappa value of 0.60 for detecting KL 0 vs. KL 1-2-3 in the knees, and 0.67 for the same in the hips, averaged across three-time points: T0, T2, T5).^41^ Furthermore, the Knee Images Digital Analysis (KIDA) technique is utilized for a more detailed quantitative analysis of Joint Space Width, Subchondral Sclerosis, and Osteophytes on knee radiographs.^42^

#### OAI

Every site used the same 3 Tesla (T) MRI scanner, ensuring consistent imaging across the OAI study. The protocol included knee, hand, and hip radiographs, and knee and thigh MRIs, aiming at comprehensive analysis. At each visit, both knees underwent radiographic evaluation using a standardized weight-bearing nonfluoroscopic fixed flexion method (SynaFlexor, Synarc, Newark, California, USA).^43^ These radiographs were centrally analyzed^44,45^ for K&L grading^46^ and OARSI Altman grading.^39^ The narrowest point of joint space in the medial part of the knee joint was determined using automated measurement software.^47^ From the MRIs, semi-quantitative scoring and quantitative cartilage measurements were performed, as well as T2-mapping and thigh muscle segmentation.

#### FNIH

Did not use additional radiographs and MRI compared with OAI. However, additional measurements were performed, including bone trabecular integrity by fractal signal analysis from knee radiographs and quantitative bone morphometry from knee MRIs.^48-50^

#### IMI-APPROACH

In the IMI-APPROACH study, bilateral weight-bearing radiography was utilized for assessing knee OA severity, focusing on joint space width (JSW) and osteophyte presence, with severity graded using the K&L system,^38^ OARSI Altman grading,^40,43^ and Verbruggen-Veys grading.^51^ The KIDA technique is utilized on knee radiographs.^42^ Advanced radiographic parameters include bone shape analyses and subchondral bone architecture assessments, highlighting OA-related bone adaptations and deformations.^52,53^ From index knee MRIs, semi-quantitative MRI scoring evaluated both cartilaginous and noncartilaginous components, covering aspects such as bone marrow edema, meniscal alteration, and synovitis.^54^ Quantitative MRI analysis evaluated cartilage volume and cartilage thickness.^47,55,56^ Furthermore, MRI was used for qualitative analysis like cartilage thickness and T2 relaxation measurements, and advanced bone shape analysis, detailing the bone area and shape changes due to OA.^53^ Lastly, whole-body CT scans and high-resolution knee CTs were performed, from which measures such as bone shape and subchondral bone architecture were evaluated, and respectively whole-body OACT grading.^55,56^

#### MOST

Radiographic evaluations encompassed bilateral, weight-bearing, fixed-flexed posterior-anterior views of the tibiofemoral joint, alongside weight-bearing lateral views of the knees. These views not only highlight the patellofemoral joint but also detail the tibiofemoral joint space. Radiographic assessments were classified using the K&L scale, ranging from 0 to 4,^38^ with an intermediary score of 3.5 indicating a grade 4 in the posterior-anterior view but showing remaining joint space in the lateral view. OARSI grading according to Altman was performed,^40,41^ with provisions for half-grade increments. The alignment of the knee was assessed using comprehensive lower limb radiographs. MRI scans of the knee were conducted for those participants without contraindications and who were within the size capacity of the ONI OrthOne extremity MRI scanner, covering 83% of the cohort. At 15 months, MRI imaging was specifically performed for a subset of participants—those experiencing knee symptoms at the 15-month checkpoint but not at the start, along with those who did not report regular symptoms at any point. Semi-quantitative MRI assessments for certain MRI-focused sub-studies were executed.^54^ In addition, the body composition of participants was analyzed using whole-body dual X-ray absorptiometry (DXA) at the outset and the 30-month mark.^57^

**Figure 5.**
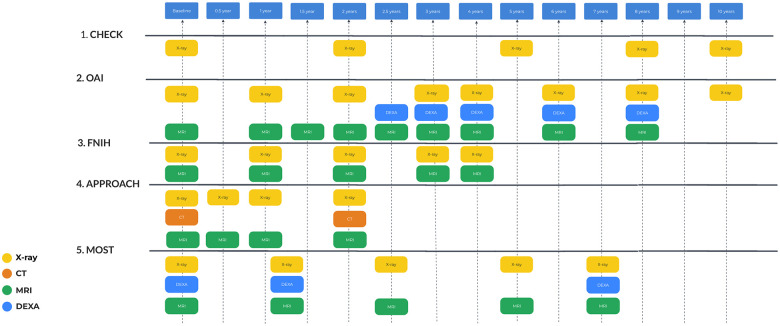
Imaging investigations schedule. No additional imaging was included in FNIH compared with OAI, but additional measurements from existing images were performed. FNIH = Foundation for the National Institutes of Health; OAI = Osteoarthritis Initiative.

All data from imaging research is present in **Table S4** and a schedule of visual investigation is in **Fig. 5**. According to these data, we can identify similarities and differences in the studies.

All studies used bilateral fixed-flexed PA views, in the case of CHECK and IMI-APPROACH according to the Buckland-Wright protocol. K&L grading and Altman/OARSI atlas were universally employed for knee evaluation across the studies, indicating a consensus on these methods for assessing osteoarthritis severity. MRI for knee evaluations frequently used semi-quantitative scoring (e.g., MOAKS, WORMS)^15,58^ and quantitative cartilage morphology (Chondrometrics),^51^ common measures across the studies that use MRI, reflecting a focus on detailed cartilage and joint analysis.

As for differences: only the OAI radiographically evaluated knee, hip, and hand joints, while CHECK used the most elaborate scorings/measurements for hips. OAI and MOST included lateral and full limb knee radiographic views, as opposed to the other cohorts that only included PA views. IMI-APPROACH is the only cohort with hand evaluations, while MOST is the only cohort with DEXA scans. Only OAI used 3T MRI imaging exclusively, while IMI-APPROACH and MOST used 1/1.5T as well. Finally, IMI-APPROACH and MOST are the only cohorts that included CT scans, evaluating specific interest in bone characteristics; IMI-APPROACH is the only cohort to include whole-body CTs.

## Discussion

Despite OA prevalence, the exact causes and mechanisms of OA remain only partially understood, prompting extensive research aimed at improving diagnosis and treatment. Several large-scale, longitudinal studies, including CHECK, OAI, FNIH, IMI-APPROACH, and MOST, have been pivotal in this research landscape, each offering unique insights into knee OA. These studies share common goals such as understanding OA’s progression, identifying risk factors, and evaluating potential interventions. Advanced imaging techniques, including MRI and radiographs, and the analysis of biochemical markers are central to these studies. These tools are crucial for diagnosing OA, monitoring its progression, and assessing the effectiveness of various treatments. The longitudinal design of these studies, which involves following participants over extended periods, is essential for capturing the natural history of OA and understanding long-term outcomes.

A consistent focus on knee OA is evident across these studies, highlighting the significant burden this condition places on individuals and healthcare systems. The knee’s susceptibility to OA due to its weight-bearing role and complex biomechanics makes it a critical target for research. Common risk factors such as age, obesity, sex, and a history of knee injury or surgery are also emphasized, underscoring the importance of identifying individuals at higher risk for developing OA. This approach aids in risk stratification and the development of targeted prevention and treatment strategies. However, there are notable differences in the design and methodologies of these studies. CHECK targets a younger demographic with early symptomatic OA, focusing on the initial stages of the disease. OAI and MOST include a broader age range and emphasize individuals with existing OA or those at high risk, providing comprehensive data on OA progression. IMI-APPROACH employs innovative machine learning techniques for participant selection, focusing on those with a high likelihood of disease progression. These differences reflect the diverse objectives and approaches within OA research.

Unique aspects of each study also contribute valuable insights. CHECK and IMI-APPROACH include assessments of hip OA, broadening the scope beyond knee OA. MOST incorporates detailed gait analysis and quantitative sensory testing, offering a deeper understanding of pain mechanisms and functional outcomes. The FNIH study, nested within the larger OAI, focuses on identifying and validating biomarkers for OA progression, emphasizing the importance of biochemical markers in understanding disease dynamics. These research efforts collectively advance our understanding of OA. They have led to the identification of novel imaging and biochemical markers critical for early diagnosis and monitoring of disease progression. Longitudinal data from these studies provide insights into the natural history of OA, highlighting patterns of structural and symptomatic changes over time. Furthermore, these studies evaluate various interventions, from lifestyle modifications to pharmaceutical treatments, offering evidence-based recommendations for managing OA.

Most cohorts focused strongly on imaging, both in acquisition and analysis. Except for CHECK, all performed elaborate MRI scans with extensive analyses, advancing OA research through quantitative cartilage morphology and semi-quantitative scores like MOAKS.^10,51,57,59^ These studies have significantly improved our understanding of OA as a whole-joint disease, highlighting cartilage thickness, bone marrow lesions (BMLs), synovitis, and meniscal pathology. In addition, cohort imaging has supported AI tool development, which requires large datasets.^60,61^ Imaging methods were largely homogeneous across cohorts, facilitating potential combination of data, though future studies should integrate newer insights, such as lower limb alignment, which has recently gained importance in OA phenotyping.^62,63^ Importantly, clinical evaluation extended beyond patient-reported questionnaires, incorporating objective knee assessments like range of motion (flexion and extension). While other joints were often overlooked, three cohorts (OAI, IMI-APPROACH, MOST) objectively measured general physical activity using accelerometer-like systems, resulting in potentially important pathology that is related to physical activity and thus complaints as experienced by patients that might be targeted in future treatment approaches.^64-66^

Despite valuable insights, findings from the studies have yet to directly impact patient care. For example, MOST linked BMLs to pain, yet trials with zoledronic acid showed no superior pain relief over placebo.^67,68^ Translating findings into clinical practice remains challenging due to OA heterogeneity, and a future step could be establishing more homogeneous cohort studies based on insights from broad, heterogeneous ones such as those highlighted here. The lack of association between pain and OA pathology limits clinical translation, and a key gap in existing cohorts is the lack of novel or objective pain measures. Only IMI-APPROACH and MOST expanded beyond traditional questionnaires (e.g., WOMAC, NRS), which provided insights into pain-structure relationships. MOST associated inflammation with pain sensitization via the more objective quantitative sensory testing, while IMI-APPROACH found MRI and radiographic OA pathologies were significantly less prevalent in patients with neuropathic-like pain (PainDETECT).^69-71^ Integrating these measures into interventional and longitudinal studies could refine patient selection and improve our understanding of pain-structure links in OA.

Several other studies, though highly valuable in the broader context of OA research, were not included in our analysis due to several reasons. Studies such as DIGItal COhort Design (DIGICOD),^72^ Musculoskeletal pain in Ullersaker STudy (MUST) cohort,^73^ Hand OSTeoArthritis in Secondary care (HOSTAS) cohort,^74^ the Rotterdam Study,^75^ Johnston County OA,^12^ and Framingham OA^76^ provide important insights into disease mechanisms, progression, and genetic risk factors. However, their datasets are not freely accessible, requiring formal collaboration or applications for data access, which limits their immediate utility for open-access research. In addition, not all these studies are focused on knee OA— DIGICOD and HOSTAS, for example, focus on hand OA, and the Rotterdam Study includes a broader epidemiological focus on aging and multiple chronic diseases. While IMI-APPROACH is also currently available only through collaboration, it has been included in our analysis as its data is expected to become open-access in the near future. Although not included here, other studies such as the UK Biobank (https://www.ukbiobank.ac.uk/), the Chingford Women’s Study,^77^ the Tasmanian Older Adult Cohort (TASOAC) study,^78^ also contain valuable information relevant to knee OA but similarly do not offer full open access and/or were not specifically designed for knee OA. Some studies, such as Johnston County OA, MUST, Framingham, Chingford, and TASOAC, are monocenter, unlike the multicenter focus of our article. In the current review inclusion was limited to multicenter, open-access, knee OA cohorts; nevertheless, the contributions of these other studies to the field of OA research remain significant, and they offer a wealth of information for future collaborative endeavors.

In conclusion, the comprehensive insights provided by these studies form a foundational understanding of OA, guiding future research and clinical practices toward more effective management strategies. By addressing OA from multiple angles, including systemic health, biomechanical factors, and early intervention, there is hope for significantly improving outcomes for individuals affected by this prevalent and debilitating condition.

## Supplemental Material

sj-docx-1-car-10.1177_19476035251326276 – Supplemental material for Comparing Five Major Knee Osteoarthritis Cohort Studies: Similarities, Differences, and Unique Aspects of CHECK, OAI, FNIH, IMI-APPROACH, and MOST Supplemental material, sj-docx-1-car-10.1177_19476035251326276 for Comparing Five Major Knee Osteoarthritis Cohort Studies: Similarities, Differences, and Unique Aspects of CHECK, OAI, FNIH, IMI-APPROACH, and MOST by Mariia Oliinyk, Simon C. Mastbergen, Anne Karien C. A. Marijnissen, David T. Felson, David J. Hunter, Micheal C. Nevitt, Harrie Weinans and Mylène P. Jansen in CARTILAGE
