# Supplementary material for: Comparing Five Major Knee Osteoarthritis Cohort Studies: Similarities, Differences, and Unique Aspects of CHECK, OAI, FNIH, IMI-APPROACH, and MOST
Source: Cartilage. 2025 Mar 19;17(3):475–89. doi: 10.1177/19476035251326276 (PMC11924058; doi:10.1177/19476035251326276)
Supplement: Supplementary file 1 [file sj-docx-1-car-10.1177_19476035251326276.docx]

**Supplementary data**

Table S1. Inclusion and exclusion criteria

|  | **CHECK** | **OAI** | **FNIH OA BIOMARKERS CONSORTIUM** | | **IMI-APPROACH** | **MOST** |
| --- | --- | --- | --- | --- | --- | --- |
| **N** | **1002** | **4674** | **194** | **406** | **297** | **3026** |
| **Inclusion criteria** | - Non-traumatic knee or hip pain or stiffness, - Aged 45–65 years old, - No previous consultation with a general practitioner, or the first consultation within 6 months before inclusion | - Overweight - Previous knee injury or surgery - Knee pain during the past year. Participants do not need to have current knee pain to take part in the study. - Parent or sibling who had knee replacement | - A Kellgren Lawrence grade (KLG) 1, 2, or 3 at baseline from central reading - Availability of knee radiograph and magnetic resonance images (MRIs) at baseline and 24 months | | - Able to walk unassisted. - ≥18 years of age. - Capable of understanding the study. - Capable of communicating in local language. - Predominantly tibiofemoral knee OA and satisfying the clinical ACR classification criteria for knee OA:   - Knee pain.   - Three or more of the following:     - >50 years of age.     - <30 min of morning stiffness.     - Crepitus on active motion.     - Bony tenderness.     - Bony enlargement.     - No palpable warmth. - High probability of progression (ranking) based on the algorithm using the following parameters:   - Reduced version of the KOOS questionnaire (pain, stiffness, and function).   - Body Mass Index.   - Numeric Rating Scale (NRS) pain^61^ of index knee at the moment of screening visit.   - NRS pain of the index knee in the last week before the screening visit.   - Age.   - Gender.   - KIDA^42^ parameters of the index knee, based on standard weight-bearing radiograph, taken at screening | - Ages 50 to 79 - Women and men - All ethnic/racial groups - Frequent knee pain, defined as knee pain on most days of the past month, reported at both screening - telephone call and about one month later at the baseline clinic visit - In those without frequent knee pain, one or more of the following risk factors for knee OA: - Overweight: above the age- and gender-specific median weight in the Framingham Study - A history of knee injury that resulted in limited ability to walk for at least 2 days - A history of knee surgery, including meniscus and ligament repair and unilateral total joint replacement |
| **Exclusion criteria** | - Any other pathological condition that could explain the symptoms (e.g. Other rheumatic diseases, previous hip or knee joint replacement, congenital dysplasia, osteochondritis dissecans, intra-articular fractures, septic arthritis, perthes’ disease, ligament or meniscus damage, plica syndrome, baker’s cyst) - Comorbidity precluding physical evaluation and/or follow-up of at least 10 years - Malignancy in the past 5 years - Inability to understand the Dutch language | - Rheumatoid arthritis - Joint replacements in both knees - Unable to walk without assistance - Unable to undergo an MRI of the knee | - Knees that were unable to meet criteria for radiographic or pain progression due to ceiling effects at baseline (minimum medial joint space width <1.0mm were excluded and/or - WOMAC pain >91 on 0-100 scale) | | - Inability to comply to the protocol. - Participation in a trial of local therapeutic intervention for index knee OA or potential systemic DMOADs at the time of inclusion, within six months before inclusion, and/or anticipated during two years of follow-up. Participation in non-interventional studies was allowed. - Surgery of the index knee in the six months before inclusion and/or scheduled or expected surgery of the index knee during follow-up. - Current pregnancy or planned pregnancy during follow-up (because of imaging). - Predominantly patellofemoral knee OA. - Secondary knee OA. For example, due to severe leg deformity (knee varus or valgus >10°), inflammatory joint disease (either autoimmune, infectious, or crystal-induced), severe chondrocalcinosis, Paget’s disease of the bone, ochronosis, acromegaly, haemochromatosis, Wilson’s disease, osteochondritis dissecans, haemophilia. - Alternative/additional causes of joint pain, for example, rheumatic symptoms due to malignancies, primary osteochondromatosis, and osteonecrosis. - Generalised pain syndrome, for example, fibromyalgia. - Patients with contraindications for undergoing MRI or CT. - Previous hip replacement or expected hip replacement within six months. - Osteosynthesis material near the knee joint. - Self-reported severe spine OA. - Current knee prosthesis; in case of surgical replacement of the index or contralateral knee during follow-up, images of that joint will be considered irrelevant and not be obtained. All other acquisitions will be performed as scheduled and patients will remain in the study. | - Unable to walk without the assistance of another person - Unable to come to clinic for the baseline examination   Total knee replacement (TKR) in both knees, or TKR in one knee with plans to have other knee replaced within the next year   - Not competent to provide informed consent - Plans to move out of the area during the next 3 years - Active life-threatening cancer or other life-threatening illness that made survival to follow up unlikely - Rheumatoid arthritis or other forms of inflammatory arthritis, based on self-report of MD diagnosis confirmed using a connective tissue screening questionnaire with high sensitivity and specificity for RA, and by self-reported use of specific medications used primarily for these forms of arthritis: e.g., gold,   methotrexate, leflunamide, plaquenil and various biologics. |

All data on Inclusion and Exclusion criteria was taken literally from study protocols.

Table S2. Questionnaires

|  | **CHECK** | **OAI** | **FNIH OA BIOMARKERS CONSORTIUM** | **IMI-APPROACH** | **MOST** |
| --- | --- | --- | --- | --- | --- |
| **Knee** | WOMAC, | WOMAC, KOOS |  | KOOS | WOMAC, KOOS, |
| **Hip** |  |  |  | HOOS | WOMAC |
| **Hand** | AUSCAN |  |  | FIHOA |  |
| **Pain** | Pain NRS, ICOAP | Pain NRS |  | Pain NRS, PainDETECT, One-month pain diary, ICOAP | Pain NRS, ICOAP |
| **Physical Activity** |  | PASE score |  |  | PASE score |
| **Quality of life** | SF-36, EQ-5D | SF-12 |  | SF-36 | SF-12, PF-10 |
| **Comorbidity** | Comorbidity list | Comorbidity Index |  | Charlson Index | Comorbidity Index |
| **Coping Inventory** | Pain Catastrophizing Scale, Pain Coping Inventory list | Coping strategies, Life space, Brief fear of movement |  |  | Coping Strategies Questionnaire, Pain Catastrophizing subscale elements, ABC Scale |
| **Depressive symptoms** |  | CES-D |  |  | CES-D |
| **Cognition** | MCS | 3MS or TICS |  |  | Fillet or Callahan 6 item screener |
| **Others** | Social Support Scale | LLDI,  ADLs.  IADLs |  |  | Pitsburg Sleep Quality Index, , Late-Life FDI |

Activities of Daily Living (ADLs); Center of Epidemiological Studies – Depression (CES-D); Functional index for hand osteoarthritis (FIHOA); Hip Injury and Osteoarthritis Outcome Score (HOOS); Instrumental Activities of Daily Living (IADLs); Intermittent and Constant OsteoArthritis Pain (ICOAP); Knee Injury and Osteoarthritis Outcome Score (KOOS); Late Life Disability Instrument (LLDI); Mental Component Score (MCS); Modified Mini-Mental State Exam (3MS); Numeric Rating Scale (NRS); Physical Activity Scale for the Elderly (PASE); Physical Functioning Scale (PF-10); Short Form 36/12 (SF-36/12); The Activities-specific Balance Confidence Scale (ABC Scale); Western Ontario and McMaster Universities Osteoarthritis Index (WOMAC)

Table S3. Biomarkers

|  | **CHECK** | **OAI** | **FNIH OA BIOMARKERS CONSORTIUM** | **IMI-APPROACH** | **MOST** |
| --- | --- | --- | --- | --- | --- |
| **Blood test** | - pAdiponectin, - pLeptin, - pResistin - sC1, 2C, - sCOMP, - sCS846, - sHA, - sOC, - sPIIANP, - sPIIINP, - sPINP, |  | - sC1, 2C, - sC2C, - sColl2-1 NO2 - sCOMP, - sCPII, - sCS846, - sCTXI - sHA, - sMMP3 - sNTXI - sPIIANP, | - S_C10C - sARGS - sC2M - sC3M, - sCOLL2_1, - sCOLL2_1NO2, - sCOMP, - sCRPM, - sCTX-I, - sHA, - shsCRP - sMMP - sNMID, - sPRO-C2, - sRE_C1M |  |
| **Urine test** | - uCTX-II, - uCTX-I, - uNTX-I, |  | - uCTXIα - uCTXIβ - uC2C - C1,2C, - uNTXI - uCTXII - uColl2-1 NO2 | uCTXII,  alphaCTX-I |  |

s-serum; p-plasma; u-urine;

Blood markers:

- ADAMTS-mediated aggrecan degradation products (sARGS)
- Bone gamma-carboxyglutamic acid-containing protein (sNMID),
- Cartilage oligomeric matrix protein (sCOMP),
- Cathepsin K-mediated type X collagen degradation fragment.(S_C10C)
- Chondroitin sulfate 846 epitope(sCS846),
- Col2-3/4 C-terminal cleavage product of human type II collagen (sC2C competitive assay in serum),
- Col2-3/4 C-terminal cleavage product of types I and II collagen (sC1, 2C),
- C-propeptide of type II collagen(sCPII),
- Crosslinked N-telopeptide of type I collagen (sNTXI)
- Cross-linked, isomerised and cathepsin K-generated fragment of type I collagen C-terminal telopeptide (sCTX-I),
- High-sensitive C reactive protein (shsCRP)
- Hyaluronic acid (sHA),
- Inflammation-related (nitrated) type-II collagen degradation fragment (sCOLL2_1NO2),
- Matrix metalloproteinase (MMP)-mediated type III collagen degradation fragment.(sC3M),
- Matrix metalloproteinase 3 (sMMP3)
- MMP-mediated C reactive protein (CRP) degradation fragment (sCRPM),
- MMP-mediated type I collagen degradation (sRE_C1M)
- MMP-mediated type II collagen degradation fragment (sC2M)
- N-MID Osteocalcin (sOC)
- N-terminal extension propeptide of type I collagen (sPINP),
- N-terminal propeptide of collagen IIA (sPIIANP),
- N-terminal propeptide of type III procollagen (sPIIINP)
- The C-terminal crosslinked telopeptide of type I collagen (sCTXI)
- Type II collagen degradation fragment (sCOLL2_1),
- Type IIB collagen propeptide (sPRO-C2),
- chondroitin sulfate 846 (sCS846)

Urine markers:

- alpha isomerised versions of the CTXI (uCTXIα)
- beta isomerised versions of the CTXI (uCTXIβ)
- Col2-3/4 C-terminal cleavage product of human type II collagen (uC2C-HUSA sandwich assay in urine),
- Col2-3/4 C-terminal cleavage product of types I and II collagen (C1, 2C),
- crosslinked N-telopeptide of type I collagen (uNTXI)
- C-terminal crosslinked telopeptide type II collagen (uCTXII),
- nitrated epitope of the α-helical region of type II collagen (uColl2-1 NO2)
- non-isomerised version of S_CTX-I (alphaCTX-I)
- urinary deoxypyridinoline and type I collagen cross-linked C-terminal (uCTX-I)

Table S4. Imaging

|  | **Joint** | **Type** | **CHECK** | **OAI** | **FNIH OA BIOMARKERS CONSORTIUM** | **IMI-APPROACH** | **MOST** |
| --- | --- | --- | --- | --- | --- | --- | --- |
| **X-ray** | Knee | Images | - Bilateral fixed-flexed (Buckland-Wright) PA - Lateral view - Skyline view | - Bilateral fixed-flexed PA - Full limb view | - No additional radiographs compared to OAI | - Bilateral fixed-flexed (Buckland-Wright) PA | - Bilateral fixed-flexed PA - Lateral view - Full limb view |
|  |  | Measures | - KL grading - Altman/OARSI atlas - Burnett grading - KIDA (joint space width, osteophyte size, subchondral bone density) | - KL grading - Altman/OARSI atlas - Joint space width - Hip-knee angle/alignment | - Joint space width - Joint space area - Bone trabecular integrity by fractal signal analysis (BTI/FSA) | - KL grading - Altman/OARSI atlas - KIDA (joint space width, osteophyte size, subchondral bone density) - Bone texture - Shape modeling | - KL grading - Altman/OARSI atlas - PF JSN - Hip-knee angle/alignment - Bone texture |
|  | Hip | Images | - Bilateral AP - Faux profile | - Bilateral AP |  |  | - DXA |
|  |  | Measures | - KL-grade grading - Altman/OARSI - Burnett grading |  |  |  |  |
|  | Hands | Images |  | - Dominant hand AP |  | - Bilateral hand AP |  |
|  |  | Measures |  |  |  | - KL grading - Altman/OARSI atlas - Verbruggen Veys scoring |  |
| **MRI** | Knee | Images |  | - 3T bilateral COR IW 2D TSE - 3T bilateral SAG 3D DESS WE (COR and AXIAL MPR) - 3T bilateral SAG IW 2D TSE FS - 3T unilateral COR T1W 3D FLASH WE - 3T unilateral SAG 2D MESE | - No additional MRIs compared to OAI | - 1.5T/3T unilateral SAG PD/IW TSE or FSE FS - 1.5T/3T unilateral AXIAL PD/IW TSE or FSE FS - 1.5T/3T unilateral COR PD/IW TSE or FSE FS - 1.5T/3T COR T1W SE - 1.5T/3T COR T1W 3D SPGR/FLASH/FFE WE or FS - 1.5T/3T unilateral SAG 2D MESE (two time points only) | - 1/1.5T unilateral AXIAL FSE PD FS - 1/1.5T unilateral SAG FSE PD FS - 1/1.5T unilateral COR STIR - 1/1.5T unilateral SAG 3D DIXON |
|  |  | Measures |  | - Semi-quantitative scoring (WORMS, BLOKS and/or MOAKS) - Quantitative cartilage morphology (Chondrometrics) - T2 mapping - Muscle segmentation | - Semi-quantitative scoring (MOAKS) - Quantitative cartilage morphology (Chondrometrics) - Quantitative bone morphometry (subchondral bone area, osteophyte volume, bone/cartilage interface signal contrast, bone shape) | - Semi-quantitative scoring (MOAKS) - Quantitative cartilage morphology (Chondrometrics) - T2 mapping - Bone shape - Cartilage surface mapping | - Semi-quantitative scoring (WORMS) |
| **CT** | Knee | Images |  |  |  | - HR knee CTs | - HR knee CTs |
|  |  | Measures |  |  |  | - Bone mineral density - Texture - Cortical bone thickness | - Bone mineral density |
|  | Whole-body | Images |  |  |  | - Low-dose whole-body CTs |  |
|  |  | Measures |  |  |  | - OACT grading |  |
